# Supplementary material for: Salmonella exploits host- and bacterial-derived β-alanine for replication inside host macrophages
Source: eLife. 2025 Jun 19;13:RP103714. doi: 10.7554/eLife.103714 (PMC12178601; doi:10.7554/eLife.103714)
Supplement: Supplementary file 1. [file elife-103714-supp1.docx]

**Strains and plasmids used in this study**

| Plasmid or strain | Genotype or description | Source |
| --- | --- | --- |
| Plasmid |  |  |
| pSim17 | temperature-induced λ-Red recombinase system; BlaS^R^ | Laboratory  collection |
| pKD3 | template plasmid containing the Cm cassette for λ-Red  recombination; Cm^R^ | Laboratory  collection |
| pKD4 | template plasmid containing the Km cassette for λ-Red  recombination; Km^R^ | Laboratory  collection |
| pBR322 | low-copy-number expression vector; Ap^R^ | Laboratory  collection |
| pMS402 | Containing a promoterless *luxCDABE* reporter gene cluster; Km^R^ | Laboratory  collection |
| pBR322-*panD* | pBR322 carrying the STM *panD* gene; Ap^R^ | This study |
| pMS402-*panD* | pMS402 carrying the STM *panD* promoter; Km^R^ | This study |
| strain |  |  |
| wild-type (WT) STM | wild-type *Salmonella* enterica serovar Typhimurium strain  ATCC 14028s | Laboratory  collection |
| Δ*panD* | WT strain *panD*::Cm; Cm^R^ | This study |
| c*panD* | Δ*panD* containing plasmid pBR322-*panD*; Cm^R^ , Ap^R^ | This study |
| ΔcycA | WT strain cycA::Cm; Cm^R^ | This study |
| Δ*fadAB* | WT strain *fadAB*::Cm; Cm^R^ | This study |
| Δ*metR* | WT strain *metR*::Cm; Cm^R^ | This study |
| Δ*leuABCD* | WT strain *leuABCD*::Cm; Cm^R^ | This study |
| Δ*leuO* | WT strain *leuO*::Km; Km^R^ | This study |
| Δ*hisABCDFGHL* | WT strain *hisABCDFGHL*::Cm; Cm^R^ | This study |
| Δ*kdpABC* | WT strain *kdpABC*::Cm; Cm^R^ | This study |
| Δ*mglABC* | WT strain *mglABC*::Cm; Cm^R^ | This study |
| Δ*potFGHI* | WT strain *potFGHI*::Cm; Cm^R^ | This study |
| Δ*znuA* | WT strain *znuA*::Cm; Cm^R^ | This study |
| Δ*argT* | WT strain *argT*::Km; Km^R^ | This study |
| Δ*gabP* | WT strain *gabP*::Cm; Cm^R^ | This study |
