# Supplementary material for: Salmonella exploits host- and bacterial-derived β-alanine for replication inside host macrophages
Source: eLife. 2025 Jun 19;13:RP103714. doi: 10.7554/eLife.103714 (PMC12178601; doi:10.7554/eLife.103714)
Supplement: Supplementary file 2. [file elife-103714-supp2.docx]

**Primers involved in this study**

| Targets | Primer Sequences (5′-3′) | |
| --- | --- | --- |
| Primers used for the construction and identification of mutant strains | | |
| Δ*cycA* | construction | F: GTATCATAGACCAAAGGCCGTAGAGCCCGCACAACACAG  ACAGGTACAGGAAGAAAAACGTGTAGGCTGGAGCTGCTTC  R: CCTCCAAATCAACGTTACGCTGTAAGCCCGGTAAGCGCC  AGCGCCACCGGGCAAAACAACATATGAATATCCTCCTTAG |
|  | identification | F: TGTGAGCTAATTCGCATTATCAAAG  R: ACGGCATTAATGAGATGATTGATGA |
| Δ*panD* | construction | F: CGGATTCGCTGGAAACCATGTCGCGGCTGATCAGCAACC  AGCCGCAGGGATAAGGACTAGTGTAGGCTGGAGCTGCTT  R: TTTCCAAAGCAGGCCACAAGCGCCTGCTTAGCCAAGGTA  AACGACAGGGTAAAGAAGTTCATATGAATATCCTCCTTAG |
|  | identification | F: CGGTTATAACGCGACCGCGATCAAA  R:GCTGCTGGAGCTGACGGAAACCAGC |
| Δ*fadAB* | construction | F: GGATTCGCTCAGTTGCCGCTGCGCTGCAATGCGAGTTATT  TAGGGGATATTATCTTTGAGTGTAGGCTGGAGCTGCTTC  R: AATACACACTTCGCTTCATCTGGTACGACCAGATCACTTT  GTGGATTCAGGAGACTGACCATATGAATATCCTCCTTAG |
|  | identification | F: AAACGGCAACTAAACTGTTTCCCGT  R: AGTGATTCCATTTTTTACCCTTCTG |
| Δ*metR* | construction | F: TAAAGCTTGGCCAGTGGGGCTGCTGATGCCAGATGGCAA  GATTAACCAGCAGTCCTGCGGTGTAGGCTGGAGCTGCTTC  R: GAAAGTCCTTCACTTCGCCATGAACAAATTGCGCTTGAG  GAATATACAGTACCTTTACACATATGAATATCCTCCTTAG |
|  | identification | F: CCATTGCAGCGTGGTTTCCGGCAGC  R: TGCAAGCCGAACTATTTAGAGTTTT |
| Δ*leuABCD* | construction | F: GAATATAATGGCAACCGGGTGATATTGCATAACCTGTAG  GCCCGGTAAGGCGTCAAGGGGTGTAGGCTGGAGCTGCTTC  R: CCGTTGCGCGGGTTTTTTTATGCCTGACGCAAGGCGCCCC  TGGAGACAAGGACCACATCCATATGAATATCCTCCTTAG |
|  | identification | F: TTTTTTATTTCGGCTGTTTCTGGAT  R: GTTGACATTAAACGGCATATCCAGT |
| Δ*leuO* | construction | F: CCAGAAAAAGGGAGTTAAGCGTGACAGTGGAGTTAAATGTG  TAGGCTGGAGCTGCTTC  R: ATAAACCAGAATTTGTTTCTGATTTATTCTGCCCGGTTCATATG  AATATCCTCCTTAG |
|  | identification | F: CATTATGAATCGCAATGGTGTGAC  R: CTCCGTCTGAATCACACCTGGT |

**Primers involved in this study (continue)**

| Targets | Primer Sequences (5′-3′) | |
| --- | --- | --- |
| Primers used for the construction and identification of mutant strains | | |
| Δ*hisABCDFGHL* | construction | F: GGCGTAAAAGTGGTTTAGGTTAAAAGGTATCAAATGAATA  AGCATTCATCGGAATTTTTGTGTAGGCTGGAGCTGCTTC  R: GATGGCTGGCATCAGGCCATCGGTTTTTTCCCAGTCCAGC  TCGCGGCGTTGTTGCTCTGCATATGAATATCCTCCTTAG |
|  | identification | F: CCTAACCAACCTAAACCGACAATTG  R: AAACGCTGTTTCGTGCGTGAGAAGA |
| Δ*kdpABC* | construction | F: GTGGGGCGGGGGCGTTTGTTCCAACAAGCGATCCGGATC  AGGACGCAAGGGTTCGTCGTGTGTAGGCTGGAGCTGCTTC  R:CTGGTTTTCTTACTTTTAGGTTATCTGGTCTATGCCCTGATTAATGCGGAGGCGTTCTGCATATGAATATCCTCCTTAG |
|  | identification | F: CAGAATGGTCAGCCCGTCAAG  R: ATACTTTTTTTACACTCCGCCC |
| Δ*mglABC* | construction | F: CCGGCGCGCAGTTTCGCTGCGCCGGGTTATCTGATAAGC  AAGGCAATAGGTCTGGATAAGTGTAGGCTGGAGCTGCTTC  R: GTGCTGAACAGCCGGGCATTTTTTTACGCTATACCCTACA  TAATAAAACCGGAGCTACCCATATGAATATCCTCCTTAG |
|  | identification | F: AAAAAGAGCACGCGTTAACACTGTA  R: AGGCTCTAAATACGCTTCGGC |
| Δ*potFGHI* | construction | F: ATGTTTTAAACCACGCCTAATGGGTTCATTTGTTAACGGAT  TTCAGAAGGAAAGCGATGGTGTAGGCTGGAGCTGCTTC  R: GAAAATGATGCCGCGACCCGCGCGGCAACGCATTGCCAT  AGTGGAAGATTTTAGTGGCTCATATGAATATCCTCCTTAG |
|  | identification | F: AAAGGCGTTTTTTAATCTGGGCTAT  R: TATTCATAATCATCAGCACATCGAG |
| Δ*znuA* | construction | F: AGATTATTAAATGCCAGGGCGACAGAGCGGGCTATCTGTT  GCACGTATTCACTTCCTCGGTGTAGGCTGGAGCTGCTTC  R: AAGTGATAGAATGTTATAATATCACATTTCACACATTCATT  ACGATGATTAGTCGCATTCATATGAATATCCTCCTTAG |
|  | identification | F: AGGGAACGAATCTCGCTTTTCTC  R: ATTCAAGCGACACGTCAGAGAGGAC |
| Δ*argT* | construction | F: CGTGATAGTTCCCCAGCGCGGCGCGTTATCCCCTTCCCGTGTA  GGCTGGAGCTGCTTC  R: GTAAAACATAAGAAAATGACGCCACTTGAGGGGTATGTCATAT  GAATATCCTCCTTAG |
|  | identification | F: CAAAGCATTCTACCTTGTCAGCCGG  R: CATACCTGCTATCTTCAACATCAGG |

**Primers involved in this study (continue)**

| Targets | Primer Sequences (5′-3′) | |
| --- | --- | --- |
| Primers used for the construction and identification of mutant strains | | |
| Δ*gabP* | construction | F: CCGGTAAACGAGCGCAATAACAATAAAGAGGTTTTAGGGTGTA  GGCTGGAGCTGCTTC  R: TTACTCCTGAGAAGCTTCCCGGCTGACGCCGGGAATAACATAT  GAATATCCTCCTTAG |
|  | identification | F: CTTTGATGAGGCGAAACAAGC  R: AATGTCATTTTTAAGCCAGCGAT |
| Primers used for the construction of complementary strains | | |
| pBR322-*panD* | F: CCCTTTCGTCTTCAAGAATTCGCTGCTGGAGCTGACGGAAAC  R: TGCGTCCGGCGTAGAGGATCCTCAGGCAACCTGTACCGGAAT | |
| pBR322 | F: GACACGGAAATGTTGAATACTCATA  R: CAAGGAATGGTGCATGCAAG | |
| Primers used for luciferase reporter system | | |
| pMS402-*panD* | F: CGAGGCCCTTTCGTCTTCACCTCGAGTCAAGGAAATCCTGGTCAATG  R: ATTTTGCGGCCGCAACTAGAGGATCCCGGGTAATCGTGAGCTGC | |
| pMS402 | F: CTGTCTCTTGATCAGATCTT  R: TTATTTCCATTACAATCAAT | |
| Primers used for RT-qPCR analysis | | |
| *16s* | F:GAAAGCGTGGGAGCAAAC  R:ACATGCTCCACCGCTTGTG | |
| *panD* | F:AAGGTTCCTGTGCCATTGAC  R:ACCGTTCACCGAGATGATTC | |
| *fadH* | F:GAAACCCACATGCCGATAAC  R:AAACTGACCGCCAATTTCAC | |
| *fadB* | F:GTTCGCTGTCTGGAAGAAGG  R: TACTGCTGCGCCATATCAAG | |
| *fadI* | F:TCTGACGCTTTTTGACATGC  R:TTCATGCAACGTTTGGGTTA | |
| *fadD* | F:AAGATCTGGCTTTCCTGCAA  R:CCCAGCTCGATAAAAAGCAG | |
| *fadE* | F:AATTGGTCGTCGTCACTTCC  R: GCGTCCTACCGACAAACATT | |
| *metR* | F:TTTGGTGATGACGTCGGATA  R:CGGGTAAATCAACAGCGTTT | |
| *hisG* | F:GTGCGTGATGATGACATTCC  R:GGAGGTGCGGATATGAGGTA | |
| *hisF* | F:TGTATTGTCGTCGGGATTGA  R:TTTTCAACTGCGTCAGATCG | |
| *leuD* | F:CCGGAATTCGTGTTGAACTT  R:GTAGAAGATGTCGGCGAAGC | |
| *leuO* | F:AGATATGGGCAAACCACAGC  R: CCTGACGGACTGAACCAAAT | |

**Primers involved in this study (continue)**

| Targets | Primer Sequences (5′-3′) |
| --- | --- |
| Primers used for RT-qPCR analysis | |
| *mglA* | F:AGCGATTAACCACGGTTTTG  R:ATTGGGTGTCGCTTTTCATC |
| *mglB* | F:AGAATCCGGTGTGATTCAGG  R:CAGCCAGGCATCCATCTTAT |
| *kdpA* | F:ACTAATCTGGCGCAGATGCT  R:ACCCTCCATGTTGACGCTAC |
| *kdpB* | F:TCGGTAAGCAGATGCTGATG  R:CCAGCGGGATCAGAAAAATA |
| *potG* | F:ATTTACGAGCATCCGACGAC  R:AGAGGCATCTGCATCGACTT |
| *potF* | F:TCTGATGGTCGTCTCTGTCG  R:GCCTTCCAGCACTTCGTTAG |
| *znuA* | F:TTAAACCGCTTGGGTTCATC  R:AACGGTTTTACATCGGCAAG |
